# Development of an mHealth App for Patients With Psoriasis Undergoing Biological Treatment: Participatory Design Study

**DOI:** 10.2196/26673

**Published:** 2021-05-10

**Authors:** Bettina Trettin, Dorthe Boe Danbjørg, Flemming Andersen, Steven Feldman, Hanne Agerskov

**Affiliations:** 1 Centre for Innovative Medical Technology Odense University Hospital Odense Denmark; 2 Department of Dermatology and Allergy Centre Odense University Hospital Odense Denmark; 3 Department of Clinical Research University of Southern Denmark Odense Denmark; 4 Private Hospital Molholm Vejle Denmark; 5 Department of Dermatology Aalborg University Hospital Aalborg Denmark; 6 Center for Dermatology Research Wake Forest School of Medicine Winston-Salem, NC United States; 7 Department of Nephrology Odense University Hospital Odense Denmark

**Keywords:** mHealth, psoriasis, biologics, participatory design, teledermatology, mobile phone

## Abstract

**Background:**

In Denmark, patients with psoriasis undergoing biological treatment have regular follow-ups, typically every 3 months. This may pose a challenge for patients who live far away from the hospital. Mobile health (mHealth) is a promising and reliable tool for the long-term management of patients with psoriasis undergoing biological treatment because the disease course can be properly monitored. Despite recent developments in mHealth, the full potential of teledermatology remains to be tapped by newer, more attractive forms of services focused on patients’ needs.

**Objective:**

This study aims to design and develop an mHealth app to support the self-management of patients with psoriasis using a participatory design.

**Methods:**

Using participatory design, we conducted 1 future workshop, 4 mock-up workshops, and 1 prototype test with patients and health care professionals to co-design a prototype. The process was iterative to ensure that all stakeholders would provide input into the design and outcome; this approach enabled continuous revision of the prototype until an acceptable solution was agreed upon. Data were analyzed according to the steps—plan, act, observe, and reflect—in the methodology of participatory design.

**Results:**

Health care professionals and patients emphasized the importance of a more patient-centered approach, focusing on the communication and maintenance of relationships. Patients perceived consultations to be impersonal and repetitive and wanted the opportunity to contribute to the agenda while attending a consultation. Patients also stated they would prefer not to attend visits in person every 3 months. On the basis of these findings, we designed an mHealth app that could replace in-person visits and support patients at in-person visits. Video consultations, self-monitoring, and registration of patient-reported outcome data were embedded in the app.

**Conclusions:**

Using participatory design facilitated mutual learning and democratic processes that gave end users a significant influence over the solution. Despite the advantages of using participatory design in developing mHealth solutions, organizational conditions may still represent a barrier to the optimization of solutions.

## Introduction

### Background

Psoriasis is a chronic, complex inflammatory disease that requires long-term management. In Denmark, patients with psoriasis receiving biological treatment have in-person follow-ups every 3 months. Patients are frustrated by these quarterly mandatory checkups and do not always benefit from consultations, which they experience as time consuming and rigidly structured, in a way that is not targeted to patients’ individual needs [1,2]. Hence, this may present an opportunity to improve the current clinical practice.

The application of telemedicine in dermatology is referred to as teledermatology (TD). TD has the potential to transform health care delivery to better meet patients’ needs [3]. TD improves access to specialist care, diagnostic accuracy, and treatment adherence while also reducing costs [4]. Attempts have been made within TD to develop *mobile* solutions, also referred to as mobile health (mHealth), using new-generation smartphones. Mobile TD has been used to treat patients with acute and chronic skin diseases [5-7]. Mobile TD may help in optimizing psoriasis treatment [8] and has been accepted by both patients and health care professionals (HCPs). It reduces in-person visits and augments person-centered care [9]. Mobile TD could be a promising tool for the long-term management of patients with psoriasis on systemic treatment (eg, biologics), where the disease course can be properly monitored and medication side effects can be detected earlier [10].

A TD solution is as effective as the in-person management of patients with psoriasis, as assessed by objective clinical outcomes [11]. TD can increase access to specialized care and reduce commuting and in-office waiting times [12]. Patients and HCPs acknowledge the benefits of telemedicine solutions; however, there are still several barriers to TD (eg, economic factors, reliability, availability, and reluctance to use it) that need to be addressed [13]. Another challenge in implementing telemedicine [14] is the limited understanding of the requirements for optimal clinical effectiveness [15]. The full potential of TD remains to be tapped by newer, more attractive forms of services that closely focus on patients’ needs [16]. One method to develop a TD solution adapted to patients’ and HCPs’ requirements is to use participatory design (PD). In PD, the focus is on designing and developing a technology that forecasts the possibilities of future technology before the solution is developed [17]. Mutual learning is the core element of PD. Through participation, the intention is to equalize the power between end users and designers by sharing knowledge. Researchers and designers need in-depth knowledge about end users’ needs and daily lives, whereas end users need knowledge about technical aspects and possibilities, together with clinical opportunities and limitations. This approach reflects the democratic aspects of PD, as it offers end users a voice in the design and development of a technology that will affect patients’ daily life and HCPs’ current clinical practice.

### Objective

The aim of this study is to design and develop a patient-centered TD solution based on patients’ and HCPs’ needs. This paper describes the design and development of an mHealth app and the involvement of patients, HCPs, researchers, and information technology (IT) designers in a PD study.

## Methods

### Overview

**Figure 1:**
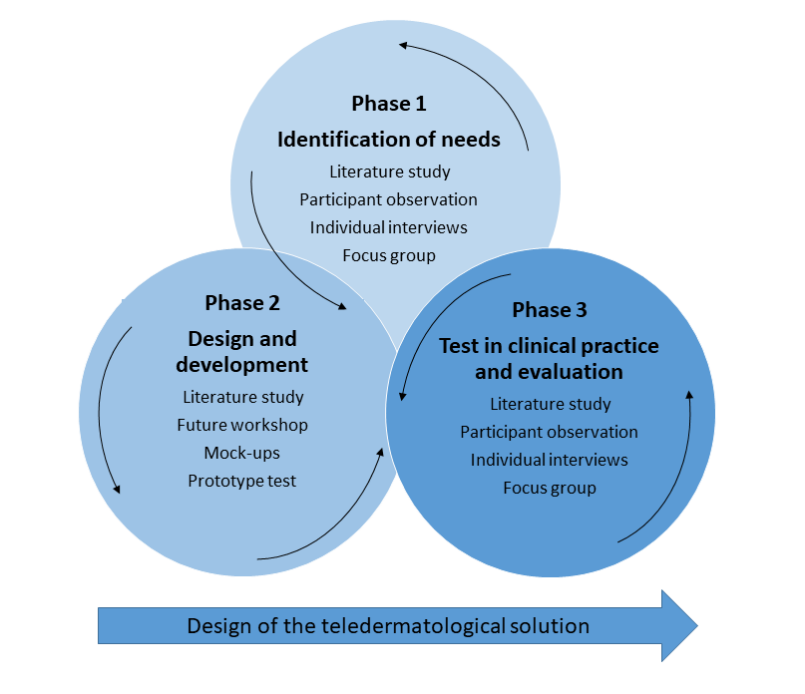
The three phases in the teledermatology solution design and development.

The PD study was conducted in 3 phases [18]. In phase 1, we identified end users’ needs by exploring their experiences. We used ethnographic methods to explore patients’ everyday life experiences with the disease and the HCPs’ experiences of clinical practice. Previous studies have reported the results from phase 1 [1,2]. In phase 2, we designed and developed a telemedicine solution to meet the needs identified in phase 1. In phase 3, we tested the prototypes in clinical practice. All phases were conducted as iterative processes throughout the study (Figure 1). Literature studies were conducted continuously in all phases to broaden our understanding of the emerging findings [19]. This paper reports reflections on phase 2 and describes and critically discusses the iterative process of the design and development of an mHealth app. In this paper, the terms high- and low-fidelity prototypes are used to visualize the design process. Design fidelity refers to the level of detail and functionality of a prototype. Low-fidelity prototypes are often created using no technology but instead a drawing, which enables the collection and analysis of feedback in the early stages of the design phase. High-fidelity prototypes are highly functional, interactive, and close to the final product [20].

### Setting

The study was conducted at an outpatient clinic of a university hospital in Denmark. Workshops and prototype testing were conducted between April and December 2019. We conducted several workshops: (1) 1 future workshop in which ideas were generated based on the needs assessment of phase 1; (2) 2 mock-up workshops with patients to discuss the initial prototype; (3) 2 mock-up workshops with HCPs to discuss the initial prototype and patients’ suggestions and corrections; and (4) 1 prototype test, where the initial prototype was tested in a real-life setting. At the time of data collection, patients were obliged to attend quarterly follow-ups to receive their biological medication, in accordance with national health policies.

### Participants

Participants in the workshops included nurses (n=9) and physicians (n=4) with several years of experience in dermatology as well as the care and management of patients with psoriasis undergoing biological treatment. Participants’ characteristics and overview of attendance are shown in Table 1. The same patients (n=3) participated in all the workshops. The patient participants were familiar with the first author, as they had contributed to their experiences of living with psoriasis in phase 1 of this study, and gave their consent to be contacted for further participation in the study. Patients were contacted by phone. In addition, a medical secretary, an IT designer, and a research team also participated. The research team consisted of a senior researcher within dermatology; a senior researcher within PD; a senior researcher within qualitative research; and the first author, who was the project leader.

**Table 1:** Participants’ characteristics and attendance at the workshops and prototype test (N=23).

Participants and characteristics	Overview of attendance		
	Future workshop	Mock-up workshop	Prototype test
Nurse, f^a^, >3 years’ experience	✓	✓	
Nurse, f, >6 years’ experience	✓	✓	✓
Nurse, f, >2 years’ experience	✓	✓	✓
Nurse, f, >4 years’ experience	✓		
Nurse, f, >13 years’ experience	✓	✓	✓
Nurse, f, >3 years’ experience	✓		
Nurse, f, >7 years’ experience		✓	✓
Nurse, f, >3 years’ experience			✓
Nurse, f, >8 years’ experience			✓
Doctor, m^b^, >20 years’ experience	✓		
Doctor, m, >5 years’ experience			✓
Doctor, f, >10 years’ experience		✓	
Doctor, f, >8 years’ experience			✓
Doctor, f, >5 years’ experience			✓
Medical secretary, f, >15 years’ experience			✓
Patient, f, aged 54 years; moderate to severe psoriasis for 31 years	✓	✓	
Patient, f, aged 28 years; moderate to severe psoriasis for 13 years	✓	✓	
Patient, m, aged 36 years; moderate to severe psoriasis for 18 years	✓	✓	✓
Information technology designer, m	✓	✓	✓
Researcher, f, experienced in participatory design	✓		✓
Researcher, m, experienced in psoriasis	✓		✓
Researcher, f, experienced in qualitative research	✓		✓
Researcher, f, PhD student	✓	✓	✓

^a^f: female.

^b^m: male.

### Data Collection and Analysis

Before the workshops and prototype testing, a detailed script describing the various steps and responsibilities was prepared and delivered to the research team and IT designers. The script included the aim of the workshops and the introduction to group exercises and plenary discussions. The study data comprised recorded transcripts from the future workshop and photographs, along with a number of written notes from all participants. Field notes taken at each mock-up workshop and the content of the discussions and suggestions for designing a prototype at the workshops were summarized in a document. Suggestions from the patients and HCPs were added to the low-fidelity prototype and served as data. During the prototype testing, all participants were given observational guides to observe and note during the test. The observational guide asked the participants to observe and note both the patients’ preparation before a consultation and the interaction during the consultation. These field notes served as data along with photographs and written field notes from one of the researchers (HA).

The analysis of the data material from the workshops and prototype test was inspired by the PD approach. The steps plan, act, observe, and reflect were followed in the data analysis in an iterative process [18]. It was not possible to plan or predict the number of iterations required to develop the final prototype. Each workshop was planned by the research group based on how the previous workshop had transpired. After each workshop, the research team shared their reflections as a part of the data analysis. On the basis of this process, the next step in the design and development phase was planned, thus facilitating mutual learning through shared experiences and perspectives. As member checking is a natural part of the PD process, participants were presented with findings from the previous activity, thus allowing them to comment on and contribute to the findings and further development of the prototype. Reporting was guided by the Consolidated Criteria for Reporting Qualitative Studies [21].

### Future Workshop

A 2-hour future workshop was conducted to identify new approaches in clinical practice through a joint critique of the existing approach [22]. The future workshop comprised 3 phases: phase 1, a critique phase; phase 2, a vision phase; and phase 3, a reality phase. In the critique phase, participants were informed about the aim of the workshop and then presented with findings from ethnographic field studies [1]. Selected findings were available in writing, together with photographs of the consultations taken during the participant observation from phase 1. The participants were then divided into 2 groups and encouraged to select the critique findings that they considered most suitable. They were also encouraged to write down additional critiques. Notes were written on post-it labels and placed on posters. Each phase of the future workshop had its own poster. In groups, they were asked to prioritize and select which points of critique they would proceed with. In the second phase of the future workshop, the vision phase, the participants were asked to convert the critique into positive ideas by asking “What if...?” They were asked to be creative and use their thoughts, visions, and dreams and to discuss categories and select the most important and significant topics. In the third phase, the reality phase, participants were asked to come up with ideas by asking “We do that by...” to create more specific and clear strategies to realize the visions. The words “What if...?” and “We do that by...” were printed on cards for the participants to fill out. The workshop was audio-recorded and further documented using notes and photos.

### Mock-up Workshops

The research group created a low-fidelity prototype, and several mock-up workshops were conducted to explore its content and detail it further. At the mock-up workshops, the aim was to further design the solution based on the results of the future workshop. The solution was an app to empower the patients. It aimed to give them the opportunity to prepare ahead of follow-up consultations and allow for video consultations. Due to organizational circumstances, it was not possible for patients and HCPs to meet at the workshops. Therefore, they were conducted iteratively with HCPs and patients separately to achieve a continuous feedback and ensure the true emancipation of PD. The various features of the app were presented to the participants, and suggestions and critiques were written down on a poster.

### Prototype Test

A high-fidelity prototype was developed based on the results from the previous workshops. As this prototype also allowed for video consultations and for patients to fill out 2 questionnaires before consultations, a prototype test was required. The 2-hour prototype test was conducted at the outpatient clinic, in the usual consultation rooms, to ensure that the setting was a realistic environment. The aim was to ensure a continued co-design with users and to test the technology. First, the participants were introduced to the prototype. They were then asked to play out a follow-up consultation based on the completed questionnaires. The set-up was planned so that it was as close to reality as possible. For this reason, doctors, nurses, and patients represented themselves. The remaining participants were equipped with observational guides and asked to make notes and write down questions, suggestions, and reflections during the consultation. Two consultations were performed simultaneously. Subsequently, the video consultations were conducted. Again, each participant represented themselves in their usual role in clinical practice, apart from one HCP, who acted as a patient, as only one patient showed up. This HCP is referred to as a patient in the following *Results* section. One video consultation was conducted during a plenary session. Those who did not participate in the consultations were asked to fill out the observational guides. The process was documented by field notes from all participants as well as photos taken by the research group. The prototype test was finalized with an evaluation of all the participants.

### Ethics

All participants received oral and written information about the study, in accordance with the applicable ethical rules [23], and gave their written consent. The Danish Data Protection Agency (2012-58-0018) approved the study.

## Results

### Future Workshop

The most common suggestion for change in clinical practice was the inclusion of a more patient-centered approach. In this approach, patients would be given the opportunity to contribute to the agenda of the consultations, which were perceived as being impersonal and repetitive. Patients expressed that they wanted to have a say in what would be discussed, rather than “just answering the same questions over and over again.”:

I know you have to inform about smoking, but don’t say it to me every time. There must be some information missing in your IT system, or something.

Patient

Patients also requested flexibility and that they would not have to attend in person every 3 months. General information regarding psoriasis and comorbidities was considered important. However, even though participants were asked about their weights or smoking habits, no strategies for how to improve, for example, lifestyle behavior, were proffered. Both patients and HCPs emphasized that the future care and management of patients with psoriasis should focus on communication and mutual relations:

Up here, I haven’t had a regular nurse, so I actually have no relationship with anyone.

Patient

Another significant issue for patients was the lack of continuity in meeting HCPs. One patient pointed out that she had been a patient at the outpatient clinic for 8 years but always met new medical doctors at consultations. The workshop further revealed that not all patients were offered the same services, for example, an appointment with a dietitian. These services or opportunities should be made visible and offered equally to all patients. This would give them the opportunity to discuss, for example, their nutrition habits with the HCP. In the reality phase of the future workshop, participants proposed that some of the abovementioned items could be offered in video consultations and prepared at home ahead of consultations:

What if maybe we only need to see them physically twice a year?

Nurse

What if the patient was given the opportunity in advance to fill in a form from home electronically [about] some areas they would like to talk about. Then you could run the consultation based on that.

Nurse

Patients also questioned having to attend in-person to collect their medication. The findings from the future workshop were depicted in a drawing, whose purpose was to serve as a design artifact for further elaboration at the mock-up workshops (for the English version, see Multimedia Appendix 1).

### Mock-up Workshops

On the basis of the results from the future workshop, it was decided to design an app for patients with psoriasis undergoing biological treatment, with the intention of meeting their needs and requests related to daily life with psoriasis and follow-up visits. HCPs acknowledged this move as a possible and appropriate solution. A low-fidelity prototype (for the English version, see Multimedia Appendix 2) was designed, and features and possible content were discussed with patients, HCPs, and an IT designer. Patients pointed out the importance of using the *good stories* to communicate knowledge and information about disease and treatment and that written knowledge and information should highlight that “you can have a good life with psoriasis.”:

I think it is insanely important that patients are also told that you can actually have or get a good life with psoriasis, it gives, like, hope.

Patient

The low-fidelity prototype included the registration of patient data, such as the self-monitoring of blood pressure, pulse, and weight. The Dermatology Quality of Life Questionnaire (DLQI) was embedded in the app. Normally, these measurements are taken at all in-person consultations. The low-fidelity prototype would make it possible for patients to complete the DLQI at home and register their patient data, meaning that this information would be automatically digitally received at the hospital. The HCPs discussed the importance of integrating the opportunity for patients to prepare ahead of consultations and that the main focus of consultations would be what is important to the patients. An existing questionnaire developed by the nurses was presented, evaluated, and adjusted by 5 new patients who did not participate in the workshops. This was done to ensure that it was also comprehensible for patients not involved in the design process. The questionnaire called preparation before consultation gave the patients several topics that could be discussed at their next consultation, including a free-text space for questions or comments. A feature in the app called preparation before consultation was designed, and the questionnaires and patient data were gathered to help patients prepare before a consultation. Furthermore, HCPs requested the development of a medication function that would allow them to order, distribute, and track patients’ medication, using a track and trace feature. Likewise, patients requested a track and trace function of their medication.

### Prototype Testing

On the basis of the suggestions, corrections, and further development, a high-fidelity prototype app called *Psoriasis* was designed (for the English version, see the Multimedia Appendix 3). It was based on an existing platform at the university hospital called *My Hospital*. *My hospital* is a digital platform developed to facilitate communication between the hospital and patients and is integrated in the patients’ personal electronic medical records. For prototype testing in December 2019, *Psoriasis* was activated in a real-life setting. In testing the preparation feature, patients were asked to fill out the self-monitoring data, the DLQI questionnaire, and the preparation before consultation questionnaire before attending a face-to-face consultation. The HCPs were instructed on how to conduct a consultation based on the feature data and responses. There was an overall agreement that whenever the questionnaire preparation before consultation was used during the consultations, it changed the content of and approach to the consultations in a positive way, by focusing on the patients’ needs and requests:

The doctor refers to a form on which the patient has noted sadness—and they talk about it. The doctor asks what the patient has been done about it and the patient says he has started seeing a psychologist. The nurse enters the dialogue and confirms the patient’s problems (has known the patient for several years). Eye contact is maintained throughout.

Field note

However, for HCPs, it was challenging to change their practice and not return to their previous routines and questions:

The doctor asks direct questions about psoriasis and treatment. They talk back and forth about the treatment and the doctor asks, “Do you have any side effects?” To which the patient replies, “No, I have a bit on my scalp.” They talk about possible treatment. The patient refers to the form and the doctor asks: “Where?” The doctor finds it and addresses what has been ticked.

Field note

One patient mentioned including a question about travel plans, as this is important due to traveling with biological medication.

In testing the video function, patients and HCPs were instructed as described above. For the video test, we used an iPad (Apple Inc) and a regular workstation in the outpatient clinic. Patients used their own devices. The functionality was good for both sound and picture. Patients were positive and expressed the personal advantages of video consultations. However, they suggested providing a guide in the app regarding how to initiate video consultations. Some HCPs were more reluctant and emphasized the need to meet and get to know patients before offering a video consultation because observing reactions, signs, and nonverbal communication was found to be challenging. Despite this, they acknowledged the patients’ perspectives.

### Final Design

HCPs and patients (including those who did not attend the prototype test) were asked to comment on the interface, usability, and content of the high-fidelity prototype. Small adjustments were made based on these comments, for example, the inclusion of more pictures to visualize the different types of psoriasis and a podcast on living with psoriasis (Table 2).

**Table 2:** Overview of the features in the app based on the identified needs.

Identified needs	Features in the app	Potential impact
Information about psoriasis and treatment	Knowledge database contains information about Psoriasis What psoriasis looks like Comorbidities Medications Video recording of how to inject oneself Video recording of emollient treatment	To support patients with psoriasis by providing them with knowledge
Information about biological treatment	Knowledge database contains information about Biological treatment Biosimilar treatment Decision on treatment start Addressing patients’ fear of discontinuance	To support patients and include them in the process of receiving biologics and encourage them in addressing their concerns
Information about living with psoriasis	Knowledge database contains information about Diet, exercise, smoking, alcohol, and stress Being together with others A podcast about living with psoriasis	To provide patients with information about lifestyle-related issues and addressing psychological aspects
Preparation before attending a consultation	Information to prepare the patients before attending a consultation, including patient data DLQI^a^ questionnaire and the questionnaire “prepare before consultation” Self-monitoring of blood pressure, pulse, weight, and urine Free-text space	To support patients in self-management, preparation before a consultation, and contributing to the agenda
Reducing in-person consultations	Video consultations for Android and IOS Guidance on how to attend a video consultation Messages to the Department of Dermatology to address nonurgent questions Information about where to pick up the biological treatment (link to map)	To support communication between the patients and HCPs^b^ and provide care and management based on the patients’ everyday life perspectives

^a^DLQI: Dermatology Quality of Life Questionnaire.

^b^HCP: health care professional.

## Discussion

### Principal Findings

In this study, an mHealth app for patients with psoriasis undergoing biological treatment was designed, adjusted, and tested through a PD process in close collaboration with patients, HCPs, IT designers, and fellow researchers. The future workshop revealed that users’ needs could be met by an app. Its use could replace in-person follow-up visits; it can be used at in-person visits and can facilitate person-centered care. The iterative process enabled us to continuously revise, redesign, and test the app until a solution that reflected the needs of the end users emerged. This highlights the importance of using PD, in which users, designers, and researchers collaborate in the design and development of new health care services. The use of PD and thus the importance of user participation and democratic processes in the medical field have been acknowledged for many years [24].

mHealth interventions have been widely used in the management of chronic conditions [25-27] and have the potential to successfully support the process. However, the use of interdisciplinary team–based approaches in the process of designing and developing mHealth solutions is essential, given that it facilitates an understanding of the context in which the solution will be used by patients and HCPs and ensures that the solution is compatible with patients’ needs and clinical demands [28].

For this study, we established a team that represented patients and stakeholders from all levels within the field, that is, the management of patients with psoriasis undergoing biological treatment. Thus, they were all familiar with the health care context and daily life with psoriasis. However, the prototype test revealed that HCPs were somewhat reluctant to use video consultations, as they were concerned about not being able to observe the patients’ nonverbal communication. Conversely, the patients experienced video consultations as suitable and convenient. This highlights not only the important democratic aspects of PD but also the shift of power dynamics in the PD process [29]. Giving patients a voice as to how they prefer the management of their condition in daily life provides HCPs with new insights and understanding, and this mutual understanding may have had a significant impact on the acceptability and implementation of the app in this study.

Likewise, the future workshop contributed to mutual learning, as it revealed that usual consultations were perceived as impersonal and repetitive. In addition, it emerged that patients would actually like to *have a say* in what to discuss and have the chance to contribute to the agenda for follow-up visits. Future workshops emphasize critique, learning, teamwork, democracy, and empowerment, which make them suitable for use in PD [22]. Future workshops were developed by Jungk [22], who believed that utopian and fantasy-based ideas and strategies for the future could be created through critique. Being creative is naturally accompanied by open mindedness; however, in practice, mental blocks often occur, thus hindering creative thinking [30]. The use of “What if...” and “We do that by...” cards in our future workshop supported the participants in being creative and share their ideas and visions for their future consultations. The use of this tool and technique engaged the participants in telling, making, and enacting [31]. Despite striving for the true emancipation and engagement of all end users, not all of the needs and requests of the end users could be met. The design and development of a technological medication delivery system have failed. Biological medication and its management are highly regulated, as this is an expensive medication that is prescribed free of charge for patients. For this reason, it was not possible to prescribe the existing web-based system in the region of Southern Denmark. However, an agreement was made among local hospitals in the region. In the future, the biological medicine will be sent to the hospital closest to the patient for distribution. The prescription and ordering of medication would continue to be made as usual on paper, which made it impossible to deliver push messages to patients when their medication had arrived. This was presented to the participants at the mock-up workshops, followed by an explanation of the existing technology and policy practices regarding biological medicine. Kyhn [32] argues that by providing end users with details about the structure and content of the emerging system, we support them in developing an understanding of the opportunities and limitations that go beyond the present interface. Therefore, mutual learning can be fostered, as we develop a shared understanding of the practice and potentials.

While engaging in a PD project, user activities are often creative and experimental, involving all stakeholders [17]. As, in our study, it was not possible for both patients and HCPs to attend a mock-up workshop at the same time, we decided to conduct several small mock-up workshops in iterative processes. The reason for this was to ensure that all stakeholders would *have a say* in the design and could influence the outcome, thereby staying true to one of the core elements of PD. Expanding the creativity in the PD process itself, for example, conducting *one-to-one* workshops, may be acceptable and may still facilitate empowerment among patients and HCPs [33].

This study was a small, single-center study connected to a clinical setting, which may put transferability in question. However, this is not an uncommon setting for qualitative research. In addition, the design process was based on findings from previous qualitative field and interview studies, thus including experiences and perspectives from other groups of patients with psoriasis and experienced HCPs. A limitation of this study was that only one patient was included in the prototype test. All 3 patients were invited, but 2 of them canceled a few hours before the test. Limited user participation is a known practical limitation in PD [34]; however, because the patients were a part of the other workshops and had planned for the mHealth solution to be tested in clinical practice, we decided to conduct the prototype test. Again, creativity and readjustment were necessary, and a HCP acted as a patient during the prototype test. To overcome this barrier, the mHealth solution was tested further in clinical practice, but it will be reported in a separate paper.

### Conclusions

Results from the future workshop, mock-up workshops, and the prototype test, based on findings from ethnographic field studies, led to the design of an app for patients with psoriasis receiving biological treatment. By using PD that facilitated mutual learning and democratic processes, end users exerted a significant impact on the solution, given that it was customized to both clinical practice and end users’ needs. The app provided both HCPs and patients the opportunity to facilitate a new approach in clinical practice. Despite the advantages of using PD in the development of mHealth solutions, organizational factors may still represent a barrier to the most desirable solution.

## Appendix

Multimedia Appendix 1 Findings from the future workshop presented as a design artifact.

Multimedia Appendix 2 Example of the paper prototype design artifact.

Multimedia Appendix 3 High-fidelity prototype used at the prototype test.

## Abbreviations

DLQI: Dermatology Quality of Life Questionnaire
HCP: health care professional
IT: information technology
mHealth: mobile health
PD: participatory design
TD: teledermatology
