# Supplementary figures and images for: Development of an mHealth App for Patients With Psoriasis Undergoing Biological Treatment: Participatory Design Study
Source: JMIR Dermatol. 2021 May 10;4(1):e26673. doi: 10.2196/26673 (PMC10501531; doi:10.2196/26673)

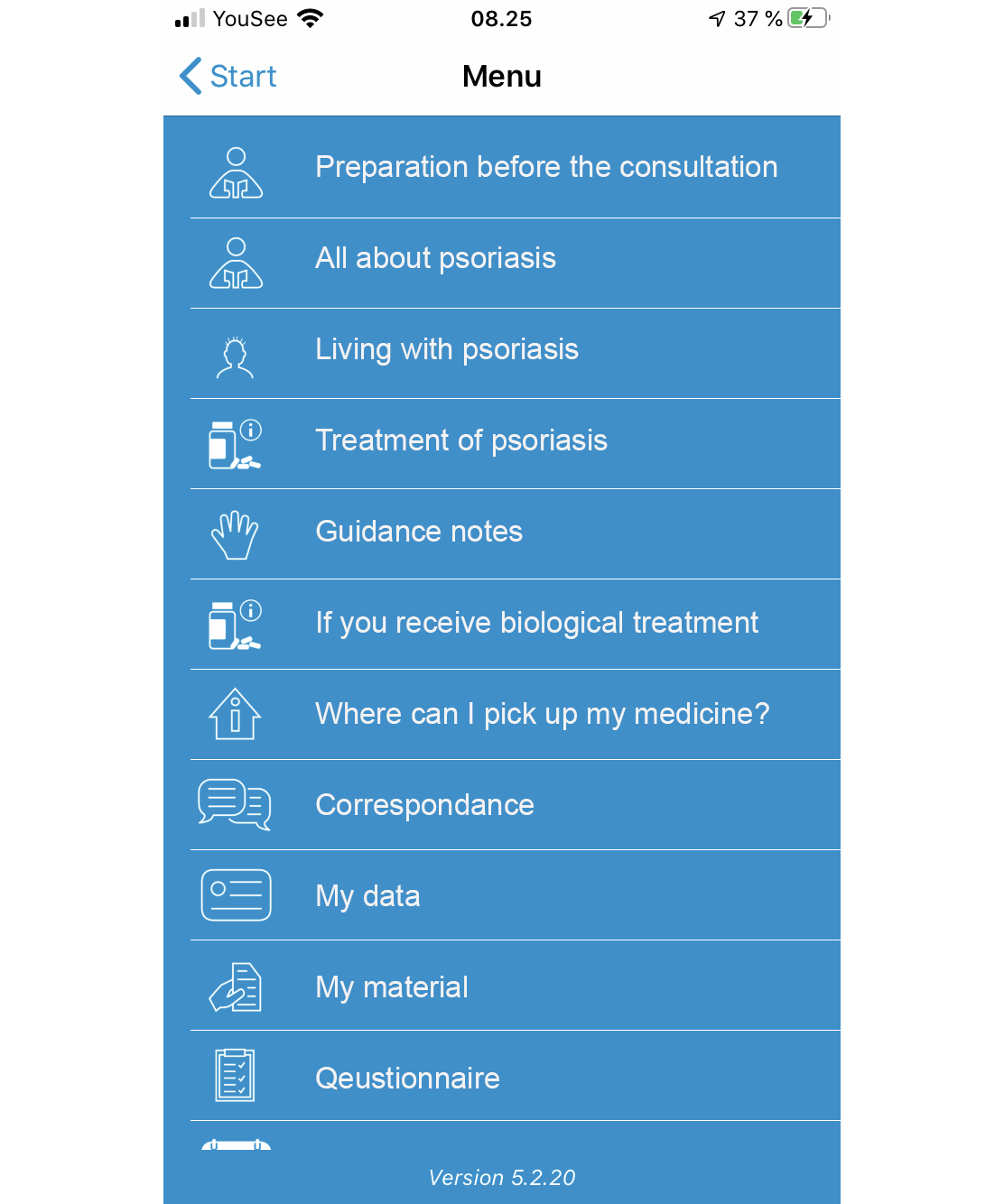

Supplement: Multimedia Appendix 3 [file derma_v4i1e26673_app3.png]
